# Applying the zoo model to conservation of threatened exceptional plant species

**DOI:** 10.1111/cobi.13503

**Published:** 2020-06-17

**Authors:** Jordan Wood, Jonathan D. Ballou, Taylor Callicrate, Jeremie B. Fant, M. Patrick Griffith, Andrea T. Kramer, Robert C. Lacy, Abby Meyer, Sara Sullivan, Kathy Traylor‐Holzer, Seana K. Walsh, Kayri Havens

**Affiliations:** ^1^ Negaunee Institute for Plant Conservation Science and Action, Chicago Botanic Garden 1000 Lake Cook Road Glencoe IL 60022 U.S.A.; ^2^ Smithsonian Conservation Biology Institute 3001 Connecticut Avenue NW Washington D.C. 20008 U.S.A.; ^3^ Species Conservation Toolkit Initiative Chicago Zoological Society 3300 Golf Road Brookfield IL 60513 U.S.A.; ^4^ Montgomery Botanical Center 11901 Old Cutler Road Coral Gables FL 33156 U.S.A.; ^5^ Botanic Gardens Conservation International, U.S. at The Huntington Library, Art Museum & Botanical Gardens 1151 Oxford Road San Marino CA 91108 U.S.A.; ^6^ IUCN SSC Conservation Planning Specialist Group 12101 Johnny Cake Ridge Road Apple Valley MN 55124 U.S.A.; ^7^ Department of Science and Conservation National Tropical Botanical Garden 3530 Papalina Road Kalāheo HI 96741 U.S.A.

**Keywords:** botanic gardens, ex situ conservation, exceptional species, metacollections, pedigree management, zoos, conservación ex situ, especie excepcional, jardines botánicos, manejo de linajes, metacolecciones, zoológicos, 植物园, 特殊植物, 迁地保护, 聚合采集, 系谱管理, 动物园

## Abstract

Maintaining a living plant collection is the most common method of ex situ conservation for plant species that cannot be seed banked (i.e., exceptional species). Viability of living collections, and their value for future conservation efforts, can be limited without coordinated efforts to track and manage individuals across institutions. Using a pedigree‐focused approach, the zoological community has established an inter‐institutional infrastructure to support long‐term viability of captive animal populations. We assessed the ability of this coordinated metacollection infrastructure to support the conservation of 4 plant species curated in living collections at multiple botanic gardens around the world. Limitations in current practices include the inability to compile, share, and analyze plant collections data at the individual level, as well as difficulty in tracking original provenance of ex situ material. The coordinated metacollection framework used by zoos can be adopted by the botanical community to improve conservation outcomes by minimizing the loss of genetic diversity in collections. We suggest actions to improve ex situ conservation of exceptional plant species, including developing a central database to aggregate data and track unique individuals of priority threatened species among institutions and adapting a pedigree‐based population management tool that incorporates life‐history aspects unique to plants. If approached collaboratively across regional, national, and global scales, these actions could transform ex situ conservation of threatened plant species.

## Abstract 2

Article Impact Statement: Using zoo‐style studbooks and pedigree management will be transformative for the conservation of exceptional plants like *Brighamia insignis*.

## Abstract (es)

Aplicación del Modelo Zoológico a la Conservación de Especies Excepcionales de Plantas Amenazadas

**Resumen**

El mantenimiento de una colección de plantas vivas es el método más común para de conservación ex situ para especies de plantas que no pueden almacenarse en bancos de semillas (i. e., especies excepcionales). La viabilidad de las colecciones vivientes, junto con el valor que representan para los futuros esfuerzo de conservación, puede estar limitada si no existen esfuerzos coordinados para rastrear y manejar a los individuos entre las instituciones. Mediante una estrategia enfocada en el linaje, la comunidad de zoológicos ha establecido una infraestructura interinstitucional que respalda la viabilidad a largo plazo de las poblaciones de animales en cautiverio. Evaluamos la habilidad de esta infraestructura coordinada de metacolecciones para apoyar en la conservación de cuatro especies de plantas curadas en colecciones vivientes en varios jardines botánicos de todo el mundo. Las limitaciones de las prácticas contemporáneas incluyen la incapacidad de recopilar, compartir y analizar los datos de las colecciones de plantas a nivel individual, así como la dificultad de rastrear la procedencia original del material ex situ. El marco de trabajo de metacolecciones coordinadas que utilizan los zoológicos puede ser adoptado por la comunidad botánica para mejorar los resultados de conservación al minimizar la pérdida de la diversidad genética que ocurre en las colecciones. Sugerimos acciones que aumenten la conservación ex situ de las especies excepcionales de plantas. Estas acciones incluyen el desarrollo de una base de datos central para acumular datos y rastrear entre las instituciones a los individuos únicos de las especies amenazadas prioritarias y la adaptación de una herramienta de manejo poblacional basada en el linaje que incorpore los aspectos únicos de la historia de vida de las plantas. Si estas acciones se plantean colaborativamente a escala regional, nacional y global, podrían transformar la conservación ex situ de las especies amenazadas de plantas.

## 摘要

保存植物活体是对不能贮存种子的植物 (特殊植物) 最常用的迁地保护方法。然而, 如果没有跨机构的生物个体跟踪和管理的协作, 活标本的生存能力及其对未来保护工作的价值可能会受到限制。动物保护团体利用基于系谱的方法建立了一套跨机构的基础工具来支持圈养动物种群的长期生存。本研究评估了这种协作性的聚合采集基础工具在支持保护四种在全球多个植物园存有活标本的植物的能力。结果表明, 当前的保护实践的局限性包括无法在生物个体水平汇编、分享和分析采集植物的数据, 以及难以追踪迁地保护植物的原始种源。植物保护团体可以采用动物园使用的协作性聚合采集框架, 通过减少采集过程中遗传多样性丧失来改善保护结果。我们建议采取行动来推动特殊植物物种的迁地保护, 包括建立中央数据库来收集数据、追踪保护机构优先保护的受威胁物种的每个个体, 以及开发基于系谱的、整合植物特有生活史信息的种群管理工具。如果在区域、国家和全球范围内进行协作, 这些行动将改变濒危植物物种的迁地保护现状。 **【翻译: 胡怡思; 审校: 聂永刚】**

## Introduction

More than 1 million plant and animal species are threatened, prompting calls for transformative changes to reverse this trend and conserve species for the public good (IPBES 2019). For many species, ex situ collections are a key component of conservation efforts, and global guidelines describe when ex situ management is recommended and how it should be conducted (McGowan et al. 2017). When an ex situ collection's purpose is extinction prevention or to support population reintroduction or augmentation, it must be managed for long‐term viability by maintaining genetic diversity and demographic security (Guerrant et al. 2004). Currently, robust policy and management practices support ex situ conservation efforts to achieve these goals for captive animal populations (Ballou et al. 2010) and for plant species that can be seed banked (Guerrant et al. 2014). However, plant species that cannot be seed banked are at risk due to a gap in infrastructure and practice (Fant et al. 2016).

As many as half of threatened plant species are exceptional; that is, they produce seeds that cannot tolerate traditional storage conditions (–18 °C, 15% relative humidity) or they produce few to no seeds (Pence 2013; Wyse et al. 2018). Consequently, for some plant taxa, the primary long‐term ex situ conservation method is to maintain them in living collections. Due to practical constraints, many botanic gardens curate only 1 or a few individuals of a species, particularly of large organisms, which greatly limits the total genetic diversity conserved (but see Griffith et al. 2015). Zoos face similar challenges, which led them to develop a robust system to manage ex situ collections as metapopulations. For species maintained at multiple botanic gardens, total potential genetic diversity held ex situ can be higher (Griffith et al. 2020), but this is often not the case because many collections are derived from the same source, or are even clones of the same plants, and may represent a limited number of unique founders (Brütting et al. 2013). With so few founders and limited collection sizes, the long‐term viability of many ex situ plant populations is questionable. This challenge of maintaining living plants in collections for conservation value is exacerbated by a lack of infrastructure to coordinate metacollections and manage them collaboratively across institutions (Griffith et al. 2020).

Unfortunately, there are currently few established best practices for maintaining viable plant populations in and across living collections, especially where the long‐term maintenance of intraspecific diversity is the primary objective (Maunder et al. 2001). The genetic diversity of an ex situ population is largely determined by the population size and the relatedness of those individuals (Lacy 1994). The founding population represents the maximum genetic diversity of an ex situ collection, after which the loss of genetic diversity through drift is inevitable unless additional founders are added. The rate of loss in genetic diversity in collections or populations is directly related to management decisions (Ballou et al. 2010). Ex situ conservation in plants has been focused on species that can tolerate long‐term storage in seed banks (Brütting et al. 2013; Guerrant et al. 2014), where the standing genetic diversity is preserved as long as seed remains viable. For species maintained as living collections, conservation practices are often focused on maintaining or maximizing the numbers of individuals, without consideration of how much of the original genetic diversity is being maintained (Ensslin et al. 2015). Consequently, collections composed of few founding individuals and grown in managed landscapes for multiple generations are at increased risk of experiencing genetic drift, inbreeding, hybridization, or selection to captivity (Havens 2004). To maintain the genetic diversity within living plant collections for the long term, strategies are needed that can manage the risk of losses over multiple generations, track the diversity of founding individuals, maximize effective population size (*N_e_*), mitigate genetic drift and inbreeding, and equalize family size to decrease the likelihood of artificial selection (Havens 2004; Lauterbach et al. 2012). Institutions with large collections will need to ensure that unintentional hybridization between related taxa is not occurring. This may involve isolating reproductive individuals, only collecting seed produced via hand pollination of bagged inflorescences, and weeding out any self‐sown individuals with unknown genetic lineages (Maunder et al. 2004). Metacollections across multiple institutions can also serve to separate different genetic lineages and as important backup collections for duplicate material.

Many in the botanical community recognize that the goal for living conservation collections, in public displays and in specialized conservation collections, should shift to long‐term population stability (Woodworth et al. 2002; Ballou et al. 2010). This is analogous to the challenges zoos faced more than 3 decades ago. Zoos recognized that most of their ex situ programs were not sustainable because they had too few animals and even fewer genetically unique founders (Lees & Wilcken 2009). Because adding new wild founders to ex situ programs was increasingly difficult, the potential for inbreeding and loss of genetic diversity compromised the long‐term viability of these captive populations (Willoughby et al. 2015). To address these challenges, zoos decided to track the pedigrees of all individuals held in ex situ collections and developed scientifically based management (e.g., breeding and transfer) recommendations to collectively manage their animals. To achieve this, they leveraged a network of international (e.g., World Association of Zoos and Aquariums) and regional (e.g., Association of Zoos and Aquariums) organizations to build infrastructure for cooperative breeding programs. This included providing infrastructure and standardized protocols to coordinate multiinstitution studbooks documenting pedigree and demographic data (Bingaman Lackey 2010) among institutions and to manage ex situ collections as a metapopulation (Table 1). With this infrastructure in place, population managers at zoos can trace lineages back to founders; quantify each individual's genetic value relative to program goals; identify and manage unequal representation of founder alleles, resulting from the overproduction of certain lineages; and increase the genetic quality of exchange between institutions, minimizing inbreeding and distributing genetic variants among institutions.

**Table 1:** Concordance between ex situ conservation infrastructure in zoos and botanic gardens^a^

Zoological infrastructure	Infrastructure available for plants?^b^	Notes
Network of accredited institutions with conservation‐focused missions and explicit, high standards of collections care and management (AZA, EAZA, ZAA)	+/–	Global network is beginning to provide accreditation (BGCI), but not to same level as zoos. Regional networks have varying agreed conservation and collections standards (ANPC, CPC)
Policies and framework to bring individuals and organizations together to conserve species ex situ (e.g., taxon advisory groups, species survival plans^1–5^)	+/–	BGCI conducts taxon‐ and region‐specific ex situ surveys^6^, PlantSearch^7^ can locate institutions curating shared species, and GardenSearch^8^ can identify expertise in different locations
Guidance on selecting species and defining ex situ program purpose and goals (e.g., integrated collection assessment and planning [ICAP] process^9^, IUCN/SSC Guidelines^10,11^)	+	IUCN/SSC guidelines^10,11^ and ICAP process^9^ can be applied to plants
System to record and disseminate information about selected species and program goals (regional collection plans^2,5,12^)	–	
Resources for animal program managers and participating institutions, including handbooks, program updates, online training modules, and contact information (AZA Animal Programs Database^13^; EAZA Population Management Online Tutorial^14^)	+/–	BGCI resources provide globally accessible and relevant resources (PlantSearch^7^, GardenSearch^8^) and national programs (CPC) provide local resources, but none focus on curating exceptional species ex situ
Guidance on collecting new founders from wild if possible^3,5,15^	+	CPC guidelines^16^
Common studbook database (ZIMS for studbooks^17^) and data‐sharing across institutions (standardized data‐entry guidelines and protocols^18–21^)	–	PlantSearch pedigree module is in development
Process to regularly evaluate genetic and demographic status of managed programs to determine if established goals are being met and to update breeding and transfer recommendations (PMx pedigree analysis software^22^) assisted by various advisory groups	–	PMxceptional is in development, but similar infrastructure is missing except at local or national levels (individual gardens, CPC); there is potential for more capacity at BGCI

a Reference‐number codes are defined in Supporting Information. Abbreviations: AZA, Association of Zoos and Aquariums; ANPC, Australian Network for Plant Conservation; BGCI, Botanic Gardens Conservation International; CPC, Center for Plant Conservation; EAZA, European Association of Zoos and Aquaria; IUCN, International Union for the Conservation of Nature; SSC, Species Survival Commissions of IUCN.

b Symbols: +, equivalent infrastructure available;–, equivalent infrastructure needed; +/–, some infrastructure available.

Such an infrastructure is currently absent in the botanical community. To test the utility of this approach to plant conservation and to lay the foundation for future work, we created pedigrees to track founder lines of 4 exceptional plant species with different life histories held in living collections (Alula [*Brighamia insignis*], a Bahamian cycad [*Zamia lucayana*], Oglethorpe oak [*Quercus oglethorpensis*], and titan arum [*Amorphophallus titanum*]). We also analyzed these data in the zoological population management software (PMx) (Lacy et al. 2012) to compare management recommendations based on pedigree to those from current botanical practices. PMx software uses pedigree data and genetic information to track founders and relatedness of the collection to provide breeding and transfer recommendations to minimize the loss of genetic diversity and avoid inbreeding within the population (Lacy et al. 2012). From these examples, we identified areas where the pedigree approach resulted in a shift in how management of collections was approached. From this work, we developed 8 actions necessary to increase the long‐term viability and conservation value of ex situ collections for threatened exceptional plant species. This work highlights how developing a zoo‐like framework for the botanical community would improve long‐term viability and conservation value of living plant collections.

## Actions Needed to Improve Management of Living Plant Collections

### Changing How Individual Plants Are Tracked Across Generations

Tracking unique founders (maternal lines) is a critical aspect of developing pedigrees. Although this has been recommended within the plant conservation community (Guerrant et al. 2004), many botanic gardens maintain plants by source (under a single accession) with little to no information of original founder or relative contribution of each founder line. This is further complicated when plants are maintained over multiple generations and maternal or paternal (pollen) lines are not tracked. Although pedigree‐based population management software, like PMx, can handle uncertainty about parentage, precision decreases as the number of possible parents increases. Without clear lineage documentation, unique or underrepresented founder alleles can easily be lost.

For example, *B. insignis* was first cultivated for ex situ management in the 1970s; additional collections were made in the 1980s and 1990s. Genetic and accession data suggest that fewer than 27 founders were brought into cultivation (Wood 2018). Attempts were made to collect unique founders, but small source population sizes increased the likelihood that founders were closely related. Of the 13 original founders brought into the National Tropical Botanical Garden (NTBG) that we were able to assign to a pedigree, 6 (46%) were lost after 40 years. This loss in diversity cannot be replenished by collection from new wild founders because *B. insignis* is possibly extinct in the wild (Walsh 2016). Some of that lost diversity may persist in other collections, but the only way to assess that is through molecular genetic study because details of relationship to original founders are largely absent.

### Creating a Centralized Database to Track Pedigrees of Ex Situ Collections

The zoo community's management strategy is to use mean kinship minimization to retain the genetic diversity of founders over multiple generations and to predict the contribution of potential progeny to the overall genetic diversity of the captive population (Ballou et al. 2010; Ivy & Lacy 2010). This is possible because zoos maintain accurate records across institutions through studbooks (e.g., ZIMS for Studbooks; Species360 2019). With these data, they can use software (e.g., PMx) (Lacy et al. 2012) that allows them to make management decisions that equalize the contribution of all founders. This requires maintaining up‐to‐date records, which is achieved through widespread participation in maintaining studbooks.

Although >1500 gardens worldwide freely contribute taxon‐level collections data to Botanic Gardens Conservation International's (BGCI) PlantSearch database, they currently do not include accession‐level and plant‐level data necessary for pedigree management. The BGCI uses PlantSearch data as the foundation for reporting progress toward the ex situ‐focused Target 8 of the Global Strategy for Plant Conservation. The BGCI and partners are working to expand this widely used tool to develop an optimal studbook‐style resource to support pedigree management of living plant collections. They have explored potential pedigree data models, are working to develop an aggregation tool for pedigree and genetic data as part of the PlantSearch database for prioritized species of concern, and anticipate launching a functional pedigree module for these taxa soon.

### Prioritizing Conservation of the Most Genetically Valuable Individuals

To maintain the genetic diversity over time, zoos use pedigrees to track descendants of all founding individuals and thus representation of founder genes in the current living population. PMx uses this information to identify the most genetically underrepresented and overrepresented individuals. For instance, of the 27 founders of *B. insignis* we identified, 3 have no living descendants in the core conservation collection at NTBG. PMx identifies such underrepresented individuals so that their propagation can be increased. Within NTBG's collection, at least 10% of individuals showed equal representation of similar founders and therefore could be removed from the population with minimal impact to the genetic diversity of the managed population. Similarly, in the case of *Z. lucayana*, a dioecious species, the entire conservation collection at Montgomery Botanical Center (Miami, Florida, U.S.A.) was derived from seed collected from 16 maternal lines. Plants from 1 of 3 subpopulations were less representative of the species’ genetic diversity (Griffith et al. 2017), suggesting that allocating resources to keeping the other 2 subpopulations would be most efficient.

Another important component of the pedigree approach is to identify overrepresentation of highly fecund individuals. Results of a study of golden lion tamarins (*Leontopithecus rosalia*) showed that most of the captive population was derived from 3 overly fecund individuals (Ballou et al. 2002), requiring a shift in breeding priorities. With plants, horticultural practices make it possible to produce many individuals from a few propagules. Although having larger numbers of plants may be desirable, if they are derived clonally or from few maternal lines, the genetic value of those plants declines. For example, ex situ collections of *Q. oglethorpensis*, an oak species endemic to the southeastern United States, were composed of only 46 individuals all from the eastern edge of its range. This number increased 3‐fold after recent expeditions to address gaps in sampling. Through these efforts, representation of genetic diversity within ex situ collections increased from 63% to 86% of the wild (source) populations. However, many new founders represent half‐siblings; consequently, some lineages are now overrepresented (Wood 2018). Using a pedigree approach, one can quantify an individual's genetic value by the amount of unique genetic diversity they represent. Because any single institution's capacity to curate numerous trees is limited, this information can be useful in the selection of which seedlings to grow to maximize genetic diversity. The pedigree analysis also highlighted that, although highly fecund accessions may appear ideal candidates for plant exchanges between gardens, these individuals may be from the same maternal lines and offer little additional conservation value to the broader ex situ population. Nevertheless, they may still be important for research and education and play a backup role if resources allow.

### Limiting Inbreeding in the Ex Situ Metacollection

The pedigree management approach can guide breeding recommendations that will minimize the potential for inbreeding in the collection. Because many accessions are composed of related plants, crosses within an institution may result in elevated inbreeding levels. Although this may have minimal short‐term impact, long‐term inbreeding can result in fitness declines. For *A. titanum*, the rarity of bloom events, clonal nature of the species, and lack of information on plant lineages contribute to increased likelihood of inbreeding in collections. To date, interinstitutional pollen exchange has been driven by availability rather than genetic planning. Some institutions have early evidence of inbreeding depression, such as nonviable seed after hand pollination (K.H., personal observation). Similarly, results of a recent study of *B. insignis* at NTBG show that pollen viability has declined in many plants, which may be evidence of inbreeding depression in this collection (Walsh et al. 2019).

### Identifying the Best Candidates for Ex Situ Transfers or Wild Reintroductions

**Figure 1:**
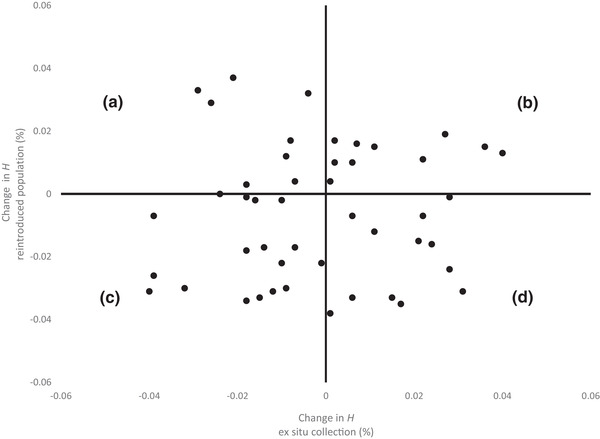
Output from population management software PMx illustrating how moving an individual (dots) from an ex situ collection to a reintroduced population (or recipient collection) can change the heterozygosity (H) (measure of genetic diversity) of the 2 groups. Individuals in quadrant (a) represent the transfer of a genetic line underrepresented in the ex situ collection and reintroduced population; transfer increases diversity of the reintroduced population (positive change in H) and reduces diversity of the ex situ collection (negative change). Individuals in quadrant (b) are overrepresented genetic lines in the ex situ collection and underrepresented lines in the reintroduced population, so transfer benefits the reintroduced population without harming the ex situ collection. Individuals in quadrant (c) represent rare genetic lines in ex situ collections but common lines in the reintroduced population, therefore, transfer from ex situ to reintroduced population has a negative impact on both. Individuals in quadrant (d) are overrepresented genetic lines in both populations, hence, transfer does not benefit the reintroduction but benefits net genetic diversity of ex situ collection by improving the balance of representation among genetic lineages.

Most botanic garden transfers are made without evaluation of how this will affect an ex situ collection's or metacollection's conservation value. Using a pedigree approach makes it possible to quantify how the selection of specimens for exchanges will affect genetic integrity of collections while allowing for the selection of individuals that would duplicate representation of unique founders in separate locations. For *B. insignis*, we used PMx to identify the genetic pros and cons of moving plants into or out of the core conservation collection at NTBG. This PMx feature can help botanic gardens determine the impacts on net genetic diversity of moving an individual between institutions; the ideal transfer increases the average genetic diversity at both the receiving and the source garden (Fig. 1). Using PMx, we identified lineages of founders missing from the core collection at NTBG and determined which individuals from the core collection could be transferred to the non‐NTBG metacollection without lowering genetic diversity at NTBG. Finally, PMx identified 3 individuals that, if lost from NTBG, would lower the NTBG collection's genetic diversity. The zoo community often uses this type of analysis to identify the best candidates for interinstitutional transfer or for reintroduction. Likewise, the botanic garden community can use it to identify individuals that will optimize the transfer of genetic diversity to the wild without compromising diversity of the core ex situ source collection.

### Using Molecular Genetic Techniques to Fill Knowledge Gaps

The pedigree approach traces all material back to the original founders to ensure that genetic diversity of those founders is maintained and represented equally throughout the metacollection. Tracking founders is difficult, especially if the species has been in collections for hundreds of years, and often requires a molecular genetic approach. The zoo community also employs genetic techniques when needed to unravel uncertain pedigree history and resolve relationships among founders (Ivy & Lacy 2010; Hogg et al. 2019). Programs like PMx can use molecular data in place of or in concert with pedigrees to make accurate metacollection management recommendations (Norman et al. 2019). In developing a pedigree for the ex situ population of *A. titanum*, it became clear that the origin of many plants is unknown. This charismatic plant has been in garden collections for more than 120 years. Seed was first collected in 1878 and shared with several institutions in Europe. Ten years later, the first inflorescence emerged at Royal Botanic Gardens, Kew (U.K.). Since the first expedition, there have been at least 20 documented introductions of new genetic material, subsequently distributed to 140 institutions (BGCI PlantSearch database). This history suggests potentially high genetic diversity within the botanical garden population, which could minimize the need for additional wild collection. Alternatively, most living plants could be descendants of only a few highly fecund individuals. Unfortunately, limited provenance information is available for most collections. Many records of wild origin are unreliable, and genetic data will be required to identify unique lineages and generate a more accurate pedigree.

### Developing Pedigree Management Software for Plants

Using the zoological community's population management software, PMx (Lacy et al. 2012), was challenging because of fundamental differences in the biology of plants, especially relative to vertebrates, which are currently the main focus of the program (Table 2). For example, most vertebrate animals have discrete male and female individuals, whereas many plants can self‐fertilize and their breeding systems vary widely. A recent version of PMx (Lacy 2012) was developed that dealt with some of these challenges (e.g., providing genetic calculations for hermaphrodites), but some software features still cannot be applied to plants or species that are managed as groups (e.g., schools of fish). Currently, the demographic component of PMx does not accommodate stage‐ or size‐based classes commonly used in plant demography. A stage‐ or size‐based model is more appropriate for plants because reproductive maturity is not necessarily related to age and many plant taxa are able to produce pollen and set seed until death. The Species Conservation Toolkit Initiative (https://scti.tools) is developing a version of PMx with improved utility for plants called PMexceptional. This new version will also benefit many animal taxa with unusual life histories or unknown paternity or that are managed in groups, including corals, many invertebrates, some fishes, and other vertebrates.

**Table 2:** Differences between animals (vertebrates) and (seed) plants that affect ex situ conservation practice^*^

Characteristics affecting conservation practice	Vertebrates	Seed plants
**Typical life‐history characteristics**		
‐ ability to assign taxon name confidently	+	–
‐ generation time	medium	short to very long
‐ number of offspring	low to medium	low to very high
‐ gender	male or female	many systems
‐ ploidy	diploid	haploid, diploid, or polyploid
**Logistical issues**		
‐ ability to move gametes	**–**(some sperm)	+ (many pollen)
‐ ability to move adults	+	– (size dependent)
‐ ability to move embryos	**–**	+ (seeds)
‐ ability to clone	**–**	+
**Current conservation protocols and investment**		
‐ existing pedigree	+	–
‐ provenance of ex situ individuals known	–	+
‐ parent determination	+	–
‐ long‐term storage ability (embryos)	–	+ (orthodox seeds)
‐ reintroduction successes	some	several
‐ likelihood of hybridization in living collections	–	+
‐ conservation status assessed	+	–
‐ cost of managing ex situ populations	+	–
‐ current conservation investment	+	–

* Key: +, relatively likely, high, or easy; –, relatively unlikely, difficult, or low.

### Conducting Outreach and Providing Education to the Botanical Garden Community

Any major changes like the ones we propose require outreach to stakeholders and ultimately their buy‐in. We have already begun discussing this approach with the botanical garden community and have a mechanism to trial the approach with a group of gardens. A new initiative led by BGCI, the Global Conservation Consortia, is trialing this approach with 4 genera: oaks, maples, magnolias, and rhododendrons. Among these genera, the most threatened and exceptional species are being prioritized for this pedigree management approach, and we envision expanding this to a few hundred species over the next several years. This approach identifies a single institution to serve as the lead so‐called species champion (analogous to a studbook keeper in zoos) to maintain the pedigree and the core conservation collection. They are also responsible for working with consortium members and safe sites, a dedicated group of other gardens and partners that acquire additional unique and duplicate material, to ensure those individuals are incorporated into the metacollection's pedigree. This trial will allow us to test the new pedigree module in PlantSearch and PMexceptional, to modify them if needed, and to develop training materials to be rolled out to the larger community. This will also facilitate the development of a core set of ex situ collection standards and a set of priority data fields to support collecting in the wild, plant records management, and, ultimately, species conservation applications.

## Prioritizing Species That Will Benefit Most From This Approach

**Figure 2:**
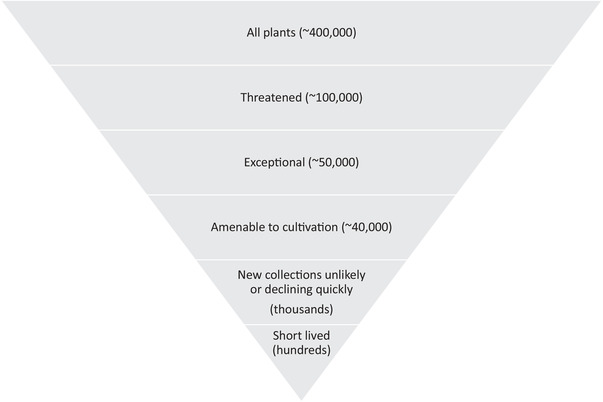
Prioritization of plant species for a pedigree management approach.

The number of threatened exceptional plant taxa that may benefit from this approach could surpass 50,000, presenting yet another challenge (Fig. 2). Identifying people and institutions to take responsibility for such a large number of pedigrees is daunting. For comparison, the zoo community manages approximately 1000 studbooks worldwide (Oberwemmer et al. 2011). Careful prioritization is needed to roll out this approach in botanic gardens for taxa that will benefit most. We suggest concentrating on the threatened taxa most at risk, for example, the critically endangered, those for which new collections from the wild are unlikely, and those in rapid decline. We also recommend focusing on species that can only be maintained as living collections, are short lived (likely to need multiple generations ex situ), and amenable to cultivation. A multitaxon assessment, such as the Integrated Collection Assessment and Planning process, can be used to further prioritize taxa if needed to maximize the overall ex situ conservation benefit (Traylor‐Holzer et al. 2019). The BGCI Global Conservation Consortia initiative is allowing us to test and fine‐tune the approach with a limited number of taxa, increasing the likelihood of success.

## Conclusions

We make the case for considering pedigree‐based management as a way to maximize the conservation value of living plant collections via studbook‐style record keeping and population management software, such as PMx. The genetic management focus of most zoo‐based breeding programs has contributed to the long‐term retention of gene diversity, helped equalize the contributions of founders, and minimized the mean kinship within scientifically managed captive populations. To achieve this, managers use several tools to compile, share, and analyze captive population data (Flesness 2003), following practices developed to maximize genetic diversity and maintain demographic stability (Ballou et al. 2010). Pedigree management and analysis tools are widely used by regional zoo associations, and management decisions are increasingly integrated across the global zoo community (Penning et al. 2009), allowing genetic diversity to be managed under a unified program. An equivalent system does not yet exist for plants, but we have identified steps and infrastructure changes that can be adapted or expanded to support it and therefore ensure the long‐term viability of ex situ collections of exceptional plant species.

## Supporting information

A key to reference codes in Table 1 (Appendix S1) is available online. The authors are solely responsible for the content and functionality of these materials. Queries (other than absence of the material) should be directed to the corresponding author.
