# Supplementary material for: Applying the zoo model to conservation of threatened exceptional plant species
Source: Conserv Biol. 2020 Jun 17;34(6):1416–25. doi: 10.1111/cobi.13503 (PMC7754355; doi:10.1111/cobi.13503)
Supplement: Supplementary file 1 — A key to reference codes in Table 1 (Appendix S1) is available online. The authors are solely responsible for the content and functionality of these materials. Queries (other than absence of the material) should be directed to the corresponding author. [file COBI-34-1416-s001.docx]

**Supporting Information**

**Appendix S1**

Reference number codes for Table 1:

^1^ AZA. 2018. *Species Survival Plan®(SSP) Program Handbook*. Association of Zoos and Aquariums, Silver Spring, MD.

^2^ AZA. 2018. *Taxon Advisory Group (TAG) Handbook*. Association of Zoos and Aquariums, Silver Spring, MD.

^3^ AZA. 2016. *AZA Policy on Responsible Population Management*. Association of Zoos and Aquariums, Silver Spring, MD.

^4^ ZAA. 2015. *Australasian Species Management Program (ASMP)*. Zoo and Aquarium Association Australasia. Retrieved from <https://www.zooaquarium.org.au/index.php/membership/policies/>.

^5^ EAZA. 2017. *EAZA Population Management Manual*. European Association of Zoos and Aquaria. Retrieved from <https://www.eaza.net/about-us/eazadocuments/#Strategies>.

^6^ BGCI. 2019. *Ex situ surveys*. Botanic Gardens Conservation International. Available at <http://www.bgci.org/plant-conservation/exsitu/>.

^7^ BGCI. *PlantSearch database*. Botanic Gardens Conservation International. Available at [www.bgci.org/plant_search.php](file:///C:\Users\khavens\Desktop\www.bgci.org\plant_search.php).

^8^ BGCI. *GardenSearch database*. Botanic Gardens Conservation International. Available at [www.bgci.org/garden_search.php](file:///C:\Users\khavens\Desktop\www.bgci.org\garden_search.php).

^9^ Traylor-Holzer K., K. Leus and K. Bauman. 2019. Integrated Collection Assessment and Planning (ICAP) workshop: Helping zoos move toward the One Plan Approach. Zoo Biology **38**: 95-105.

^10^  IUCN/SSC. 2014. *IUCN Species Survival Commission Guidelines on the Use of Ex situ Management for Species Conservation, Version 2.0.* (Gland, Switzerland: IUCN Species Survival Commission.).

^11^ McGowan P.J.K., K. Traylor-Holzer and K. Leus. 2017. IUCN guidelines for determining when and how ex situ management should be used in species conservation. Conservation Letters **10**: 361-366.

^12^ Smith B., M. Hutchins, R. Allard and D. Warmolts. 2002. Regional collection planning for speciose taxonomic groups. Zoo Biology **21**: 313-320.

^13^ AZA. 2019. *Animal Programs Database Association of Zoos and Aquariums.* Available at <https://www.aza.org/about-animal-programs-database>.

^14^ EAZA. 2019. *Population Management Online Tutorial European Association of Zoos and Aquaria.* Retrieved from <https://rise.articulate.com/share/Uqa1mUwqIn66HIeecF3_rWpXmKKqdyNf#/?_k=70ytav>.

^15^ ZAA. 2015. *Policy – Animal Transactions (ASMP).* Zoo and Aquarium Association Australasia. Retrieved from <https://www.zooaquarium.org.au/index.php/membership/policies/>.

^16^ Maschinski J. and M.A. Albrecht. 2017. Center for Plant Conservation's Best Practice Guidelines for the reintroduction of rare plants. Plant Diversity **39**: 390-395.

^17^ Species360. 2019. *Zoological Information Management System (ZIMS) for Studbooks*. Species360. Available at zims.Species360.org.

^18^ Earnhardt J.M., S.D. Thompson and G. Tuner-Erfort. 1998. *Standards for Data Entry and Maintenance of North American Zoo and Aquarium Animal Records Databases*. (Lincoln Park Zoo).

^19^ AA. 2010. *Amphibian Data Entry Guidelines.* Amphibian Ark. Available at <http://www.amphibianark.org/pdf/Amphibian-Data-Entry-Guidelines-2010.pdf>.

^20^ Wilcken J. and C. Lees. 2012. *Managing Zoo Populations: Compiling and Analysing Studbook Data*. Australasian Regional Association of Zoological Parks and Aquaria, Mosman.

^21^ WAZA. 2018. Resource Manual for International Studbook Keepers. World Association of Zoos and Aquaria. Retrieved from <https://www.waza.org/priorities/conservation/international-studbooks/isb-resource-manual/>.

^22^ Lacy R.C., J.D. Ballou and J.P. Pollak. 2012. PMx: software package for demographic and genetic analysis and management of pedigreed populations. Methods in Ecology and Evolution **3**: 433-437.
